# Enhancing citrus fruit yield investigations through flight height optimization with UAV imaging

**DOI:** 10.1038/s41598-023-50921-8

**Published:** 2024-01-03

**Authors:** Soon-Hwa Kwon, Ki Bon Ku, Anh Tuan Le, Gyung Deok Han, Yosup Park, Jaehong Kim, Thai Thanh Tuan, Yong Suk Chung, Sheikh Mansoor

**Affiliations:** 1 Citrus Research Institute, National Institute of Horticultural and Herbal Science, Rural Development Administration, Jeju, 63607 Republic of Korea; 2 Department of Plant Resources and Environment, Jeju National University, Jeju, 63243 Republic of Korea; 3 Department of Practical Arts Education, Cheongju National University of Education, Cheongju, 28690 Republic of Korea

**Keywords:** Plant breeding, Plant sciences, Plant physiology

## Abstract

Citrus fruit yield is essential for market stability, as it allows businesses to plan for production and distribution. However, yield estimation is a complex and time-consuming process that often requires a large number of field samples to ensure representativeness. To address this challenge, we investigated the optimal altitude for unmanned aerial vehicle (UAV) imaging to estimate the yield of *Citrus unshiu* fruit. We captured images from five different altitudes (30 m, 50 m, 70 m, 90 m, and 110 m), and determined that a resolution of approximately 5 pixels/cm is necessary for reliable estimation of fruit size based on the average diameter of *C. unshiu* fruit (46.7 mm). Additionally, we found that histogram equalization of the images improved fruit count estimation compared to using untreated images. At the images from 30 m height, the normal image estimates fruit numbers as 73, 55, and 88. However, the histogram equalized image estimates 88, 71, 105. The actual number of fruits is 124, 88, and 141. Using a Vegetation Index such as I_PCA_ showed a similar estimation value to histogram equalization, but I_1_ estimation represents a gap to actual yields. Our results provide a valuable database for future UAV field investigations of citrus fruit yield. Using flying platforms like UAVs can provide a step towards adopting this sort of model spanning ever greater regions at a cheap cost, with this system generating accurate results in this manner.

## Introduction

Among the most significant non-climacteric tropical fruits in the fruit business are citrus fruits, which are consumed worldwide. This is due to their very pleasant flavor and a wide range of nutritional advantages^1,2^. Citrus phytochemicals, such as phenolics, flavonoids, limonoids, carotenoids, and volatile terpenes, have been linked to a lower risk of a number of health issues in several randomized animal and clinical studies^3,4^. These phytochemical profiles, nevertheless, are reliant on a number of variables, including citrus type, growing conditions, and fruit development^5,6^. *Citrus unshiu* is cultivated primarily on Jeju Island in Korea, Southeast China, and Japan. The characteristic of *C. unshiu* fruit is seedless and easy to peel. The fruit is rich in vitamins, flavonols, and anti-inflammatory and antioxidant properties that can protect against various diseases^7,8^. It is a very popular citrus fruit in Korea that is consumed as fruit or juice. Smart farming is vital for agricultural sustainability^9–11^, and remote sensing has been a useful tool in these efforts^12^. In addition to other requirements, remote sensing has proven crucial for soil management^13,14^ insect control^14,15^, weed identification^16–19^, and vegetation health and vigor^20^. The development of precise ways to identify and count individual trees from high-resolution optical imaging has been a key research area for the effective management of tree plantations and orchards. It is possible to anticipate yields more accurately, comprehend tree growth traits, and spot abnormalities in tree growth by combining spectral data from individual tree canopies with field data^21,22^. The unmanned aerial vehicle (UAV) for yield estimation has become popular recently due to its efficiency^23^. The RGB image is usually used, and a new approach that integrated UAV-based vegetation index (VI) and abundance information obtained from spectral mixture analysis (SMA) was established to improve the estimation accuracy of yield estimation^23^.

The UAV image analysis for yield estimation is one possible method that increases efficiency. There are two estimation methods. The one uses alternative parameters such as geometric traits and vegetation indices. These alternative methods are usually used when the fruit is hardly distinguished from the tree or increases the efficiency of the estimation. For example, the canopy projected area and canopy perimeter are correlated with the fruit load of peach trees^24^ the individual crown area of the olive or almond can be used to predict yield^25,26^. The other one is directly counting the fruit from the tree. In the case of *C. unshiu*, using direct counting methods is easy to approach because of the fruit color. The color of the *C. unshiu* fruit peel is orange, distinguished from the green leaf of a tree. For the *C. unshiu* fruits, the peel is green that accumulates β, ε-carotenoid at the primary time; by the time flow, the peel accumulations change to β, β-carotenoid that color is orange^27–29^. The image analysis can be separated from the tree and counted using the color difference between the leaf and fruit.

Counting fruit from *C. unshiu* tree image by the algorithm is already been studied and proven efficient^30^. Also, it was suggested that using UAV is a kind of remotely sensed real-time quantification, which is good for optimizing resource utilization^31^. Therefore, for predicting the yield of *C. unshiu,* using a UAV aerial image is one of the effective methods. The possibility of this method was reported on a limited basis that tested only 15 m flying height^32^. Even there are few references for the flying height of UAVs and image analysis. Additionally, which image analysis methods are proper for fruit yield prediction have barely been studied. Histogram equalization is the method that improves the contrast of the image for medical image analysis or other fruit-counting studies^33–36^. For farmers’ profit and to reduce resource waste, accurate and adequate predicting of yield is essential. Also, decreasing the effort, such as human labor or time consumption, is emphasized during the data collection for estimating the final yield. For example, in data collection, researchers in Korea estimate fruit yield every August by manually dispatching and counting the fruit from 640 trees across 320 orchards. This approach is very inefficient and produces many errors. To solve these waste and inefficient, the appropriate application of technology affects the efficiency of yield prediction, would be resulting in an increment in profit for farmers and consumers and decrement in resource waste. In the current study, we compared the five flying heights of UAV images of *C. unshiu*. Also, we compared normal images to histogram equalization images in which flying height and analysis methods were used.

## Materials and methods

### Study area and data collection

The study areas are 1821-1, OraI-dong, Jeju-si, Jeju-do, the Republic of Korea as area A and 94, Haryegwangjang-ro 41beon-gil, Namwon-eup, Seogwipo-si, Jeju-do, the Republic of Korea as area B. The area of each field is 1500 m^2^ and 1200 m^2^. The image of a field-planted *C. unshiu* tree was taken by unmanned aerial vehicles (UAV) as the Matrice 300 (SZ DJI Technology Co., China), the UAV which mounted RGB camera (X5S, SZ DJI Technology Co., China), and multispectral image sensor (Rededge M, Micasense) record the image of trees at each height. The FOV of UAV is 145°, and the image sensor size is 13 mm × 17.3 mm. The flight height of the UAV for taking the plant image was 30 m, 50 m, 70 m, 90 m, and 110 m. The image used in the current research was taken in 2021.5.7.

### Image collection and analysis

The entire steps were processed in MATLAB. First, the histogram equalization was pre-processed by the histeq () command to enhance the contrast of the images. Next, the equalized RGB images obtained through the UAV were converted into HSV images by rgb2hsv() syntax, and the area of the citrus trees was separated from the ground using ${I}_{PCA}$ (principal component analysis index) (Eq. 1).

1 $${I}_{PCA}= 0.994|R-B|+0.961|G-B|+0.914\left|B-R\right|.$$

$$\left[ R,\,G, \,and\, B \,are \,Red, \,Green, \,and \,Blue\, channel \,of\, histogram\, equalized \,image \right].$$

In addition, color thresholding was applied to obtain the area of the citrus and obtain the corresponding image. The upper and lower HSV thresholds are (19, 31, 221) and (39, 243, 255), respectively (Eq. 2).

2 $$Area\, of\, the\, Citrus={H}_{upper}<HSV\left(:,:,1\right) \& HSV\left(:, :, 1\right)<{H}_{lower}\dots {\& S}_{upper}<HSV\left(:,:,2\right) \& HSV\left(:, :, 2\right)<{S}_{lower}\dots \& {V}_{upper}<HSV\left(:,:,3\right) \& HSV\left(:, :, 3\right)<{V}_{lower}.$$

The tree area and citrus area overlapped, color was applied only to the areas overlapping with the tree, and the number of pixels was counted. The same process was repeated for all altitudes.

In order to use a vegetation index to detect fruits, VI methods (I_PCA_, CIVA, I_1_) were applied to the original image (Eqs. 1, 3, 4)^37^.

3 $${\text{CIVA }} = \, 0.{\text{441R }} - \, 0.{\text{811G }} + \, 0.{\text{385B }} + { 18}.{78},$$

4 $${\text{I}}_{{1}} = {\text{ R }} + {\text{ G }} - {\text{ 2B}}.$$

### Statistical analysis

**Figure 1:**
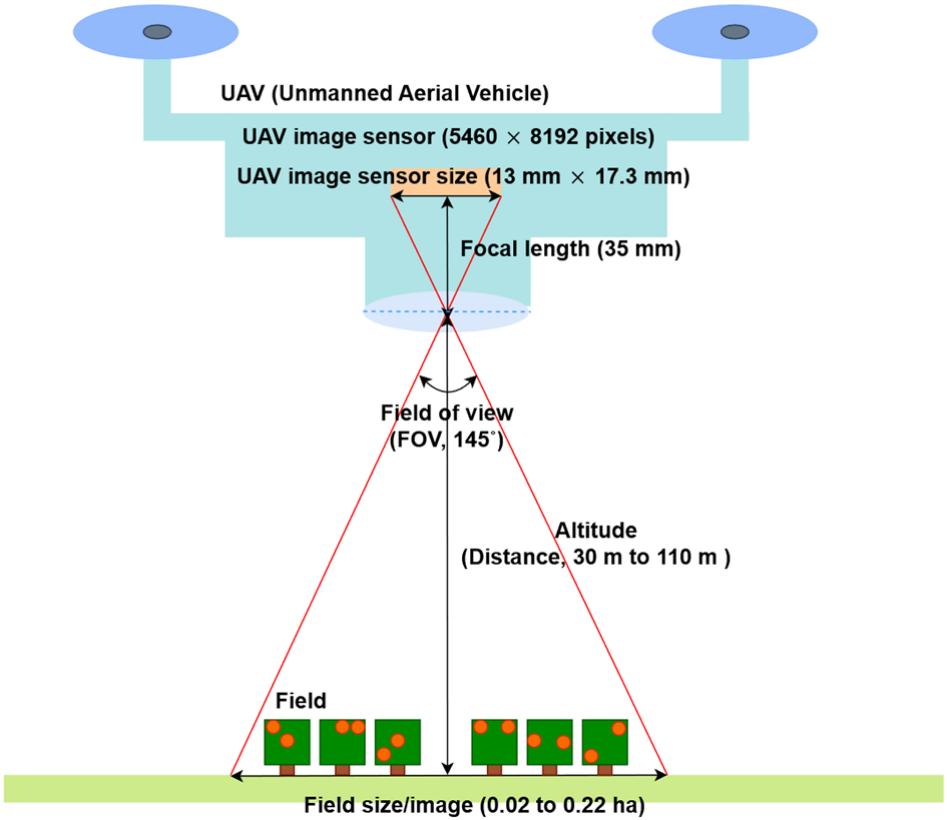
The relation of UAV image sensor size, focal length, field of view, and fling altitude.

Microsoft Excel and R software analyzed the data from images. The data organization and basic calculations, such as unit change, were conducted in Excel. For the calculation of the actual size of the field in a picture, Eq. (5) was used (Eq. 5). Besides, to estimate the fruit number in a tree, Eq. (6) was used (Eq. 6). The average diameter of the *C. unshiu* was confirmed by Jeju’s special self-governing province agricultural promotion agency as 46.7 mm, and the average fruit area was 17.12 cm^2^/fruit^38^. The detailed position and size of the sensor, focal length, and altitude are represented in Fig. 1.

5 $$Real\, size\, per\, image= \frac{UAV\, flight\, altitude \times Sensor\, size (13\text{ mm} \times 17.3\text{ mm})}{Focal\, length\, of \,UAV (35\text{ mm})},$$

6 $$Estimate\, fruit \,number= \frac{Number \,of \,fruit \,pixel\, at \,each\, altitued \,image}{Average\, pixel\, per\, fruit\, at \,each \,altitude}.$$

Before the comparison, it confirmed that the data satisfied the t-test assumption; the data from 90 and 110 m flight altitudes did not satisfy it. Therefore, R software conducted the t-test for 30 m, 50 m, and 70 m and the Wilcoxon rank-sum tests for 90 m and 110 m.

## Results

**Figure 2:**
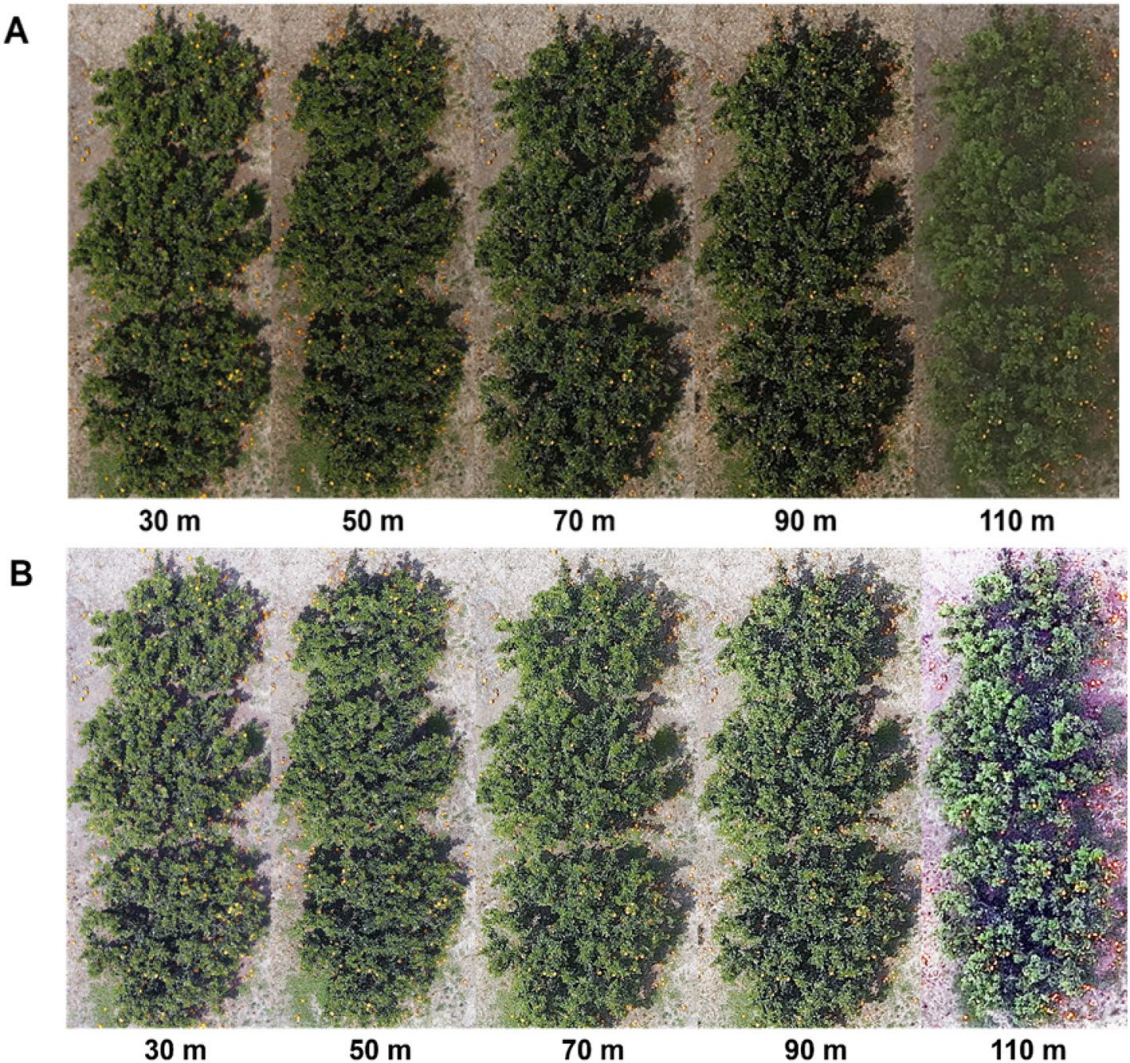
Image of each height (30 m, 50 m, 70 m, 90 m, 110 m) of *C. unshiu* trees. (**A**) Untreated RGB image; (**B**) histogram equalized RGB image.

As shown in Fig. 2, the aerial photographs were collected at various altitudes (30 m, 50 m, 70 m, 90 m, and 110 m). The fruits may be identified by their small yellow spots with just the bare eyes. Figure 2A,B show a regular RGB image and an RGB image that has been histogram-equalized for comparative purposes. The characteristics of each altitude picture are shown in Table 1. The 110 m flying altitude image (5460 × 8192 pixels) comprises 0.22 hectares per image, with 2 pixels roughly equivalent to 1 cm^2^ of objects. The image taken from a height of 30 m, in comparison, comprises 0.02 ha per image; to depict an item that is 1 cm^2^ in size, one needs around 27 pixels. In other words, photographs taken from high altitudes include a lot of information from a large area. Unfortunately, a low-flight altitude image’s narrow field of view prevents it from gathering much information from the region that was acquired, despite the fact that the image is still distinct and detailed. According to the data, a *C. unshiu* fruit requires 34 pixels for a picture taken from 110 m above sea level and 464 pixels from 30 m above.

**Table 1:** Image characteristics of RGB images from various UAV altitudes.

UAV flying altitude (m)	Real area/image (ha/image)	Pixels/cm^2^ value from image	Pixels/fruit value from image^a^
30	0.02	27	464
50	0.05	10	167
70	0.09	5	85
90	0.15	3	52
110	0.22	2	34

^a^Annual average citrus fruit size of 2021 (46.7 mm) was used for these values calculation as a constant.

**Figure 3:**
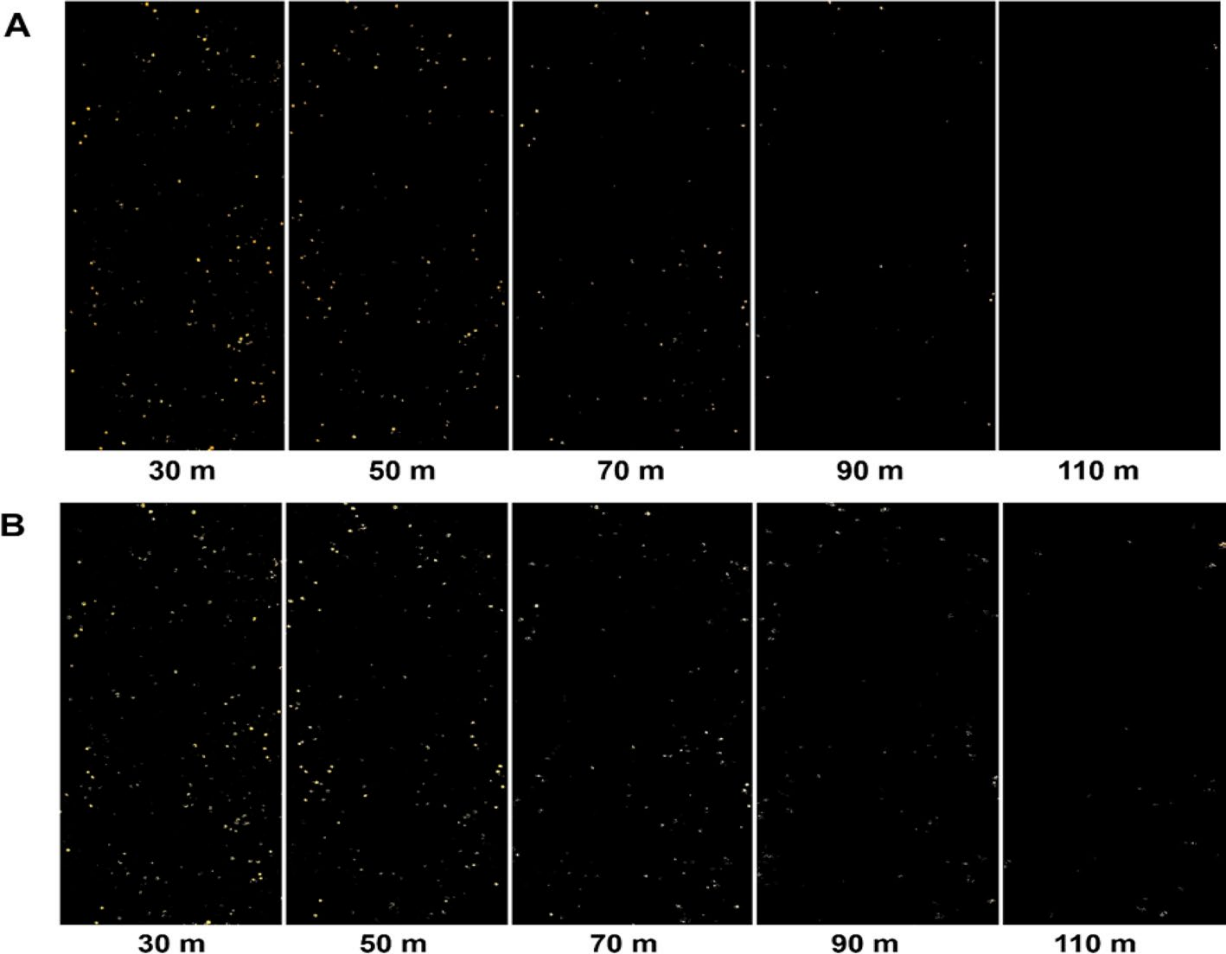
Image of each height (30 m, 50 m, 70 m, 90 m, 110 m) of *C. unshiu* fruit detected from trees. (**A**) Fruit from untreated RGB image; (**B**) fruit from histogram equalized RGB image.

The detail of the image affects fruit detection. Tables 2 and 3 represent the number of fruit pixels in the image and fruit estimation from each tree. Table 2 shows the results from a normal RGB image. At a low altitude, successfully distinguished and detected fruit from the tree. However, the high altitude fails to distinguish fruit from the tree. In the image from a flight height of 110 m, tree number 2 and 3 represent no fruit in the tree at analysis. Table 3 shows the results from the image after the histogram equalization. It can detect more fruits than a normal image; the detected fruit is increased by approximately 10%, and it can detect fruit from a 110 m altitude image, which fails to detect fruit in the normal version. Figure 3 displays the fruit detected in the images from each altitude. The image at a 30 m altitude showed more fruit than the images captured at other altitudes (Fig. 3A,B). Additionally, the histogram-equalized images detected more fruit than the untreated images at high altitudes such as 90 m and 110 m (Fig. 3A,B).

**Table 2:** Image analysis results from the normal RGB image.

UAV flying altitude (m)	Tree number	Number of fruit pixels in the image	Estimate fruits number
30	1	33,667	73
	2	25,301	55
	3	41,256	89
50	1	6558	39
	2	5160	31
	3	7050	42
70	1	1338	16
	2	876	10
	3	1704	20
90	1	360	7
	2	279	5
	3	231	4
110	1	42	1
	2	0	0
	3	0	0

**Table 3:** Image analysis results from applying histogram equalized image.

UAV flying altitude (m)	Tree number	Number of fruit pixels in the image	Estimate fruits number
30	1	40,818	88
	2	32,964	71
	3	49,008	106
50	1	11,073	66
	2	8511	51
	3	8919	53
70	1	2436	29
	2	1674	20
	3	2895	34
90	1	1212	24
	2	546	11
	3	897	17
110	1	243	7
	2	18	1
	3	228	7

Applying histogram equalization to the image is statistically significant at lower flight altitudes (30 m and 50 m), while relatively high altitudes (70 m, 90 m, and 110 m) show no statistically significant. Table 4 shows that at the flight altitudes of 30 m and 50 m, the p-value is lower than 0.05 at the estimated fruit number. However, at the number of fruit pixels in the image, the image from a 30 m altitude is not significantly different between the normal and histogram-equalized images.

**Table 4:** T-test and Wilcoxon rank sum test results between normal images and histogram equalized image.

UAV flying altitude (m)	Number of fruit pixels in the image			Estimated fruit numbers		
	Normal image	Histogram equalized image	p-value	Normal image	Histogram equalized image	p-value
30^a^	33,208.00 ± 4607.63	40,930.00 ± 4631.84	0.31^ns,b^	72.05 ± 12.17	88.27 ± 12.23	0.31^ns^
50	6256.00 ± 566.11	9501.00 ± 794.78	0.03*	37.48 ± 4.15	56.92 ± 5.83	0.03*
70	1306.00 ± 239.56	2335.00 ± 356.07	0.08^ns^	15.34 ± 3.45	27.42 ± 5.12	0.08^ns^
90	290.00 ± 37.64	885.00 ± 192.35	0.10^ns^	5.63 ± 0.89	17.18 ± 4.57	0.10^ns^
110	14.00 ± 14.00	163.00 ± 72.63	0.18^ns^	0.41 ± 0.50	4.73 ± 2.58	0.18^ns^

^a^T-test was used at 30 m, 50 m, and 70 m because the data satisfied the T-test assumption. The Wilcoxon rank-sum test was used for comparison at 90 m and 110 m data that did not satisfy the t-test assumption.

^b^NS, nonsignificant at p > 0.05, *significant at 0.05, and **significant at 0.01.

The results of additionally applied various vegetation indexes such as I_PCA_, CIVE, and I_1_ at 30 m altitude are in Table 5. Also, the actual yield of citrus fruits from each tree is represented. In the case of actual yields, the fruits hidden by the leaf are added so that the number is larger than our estimate. Nevertheless, the estimates using the histogram equalized image are similar to the actual yields among the images used in this comparison. Also, using the VI I_PCA_ image showed a similar estimation level to the histogram equalized image. In contrast, applying VI I_1_ represents a gap from the actual yield that is inappropriate for estimating fruit yield.

**Table 5:** Compare the actual yield of three citrus trees and estimate methods by various images (Normal RGB image, Histogram equalized image, VI I_PCA_, CIVE, and I_1_ applied images).

UAV flying altitude (m)	Tree number	Actual yield	Normal image	Histogram equalized image	Applied VI I_PCA_ image	Applied VI CIVE image	Applied VI I_1_ image
30	1	124^a^	73	88	87	85	28
	2	88	55	71	71	68	24
	3	141	89	106	105	101	30

^a^Actual yield from each citrus tree.

## Discussion

In recent years, UAVs have drawn a lot of attention to measuring secondary traits, such as plant height and spectral reflectance, in a large area. This is due to the benefits of UAVs, which include their ease of operation, highly flexible and timely control, super-high spatial resolution, and ability to quickly retrieve large amounts of field data due to a reduction in planning time. A UAV may be fitted with a variety of sensors, including multispectral and RGB cameras, which are valuable in agricultural applications. Also, the development of inexpensive UAVs and image sensors has made UAVs a hot topic in the realm of agricultural remote sensing. In particular, UAVs provide an entirely new perspective to the agricultural landscape by collecting remote sensing data at very low altitudes. The literature on the use of UAV image collection and analysis for ecological applications, natural resource monitoring, and agricultural management has exploded during the past 10 years^39–42^. There is a growing body of literature on the use of UAVs in agriculture, notably in the field of precision agriculture. UAV workflows are being added to agricultural management in order to precisely observe, measure, and monitor crop conditions throughout the growing season^43–46^ estimate and measure crop yields^47,48^. The fundamental job of feature (or object) extraction is central to these requirements. Globally, the gathering of UAV photography for agricultural applications is growing, and more of these unique examples are required to create more standard procedures that will aid field and research managers in managing vast amounts of high-quality images. Ground sample distance (GSD), which varies depending on flight height and camera, may vary from a few centimeters^22^ to considerably greater in UAV-collected pictures. The image sizes at these high resolutions will accumulate. For instance, a 4-band picture mosaic of a 100 ha (250 acres) study area at 10 cm GSD may be only moderately large (e.g., 0.5 Gigabyte), but the entire data package, which includes all of the input photographs and the final photogrammetric outputs, may be as large as 5 Gb.

Uavs are currently gaining popularity for monitoring purposes and their use in agriculture. Not only in agriculture but also in other crucial fields like power-line inspection, pipeline monitoring, construction, structural monitoring, etc., UAVs are becoming more and more common for monitoring^49^. The Dronfruit initiative was created under the context of the Andalucia Region’s 2014–2020 Rural Development Program, with funding from the Agricultural European Innovation Partnership (EIP-AGRI). EIP-AGRI was established in 2021 to promote sustainable farming and forestry that produces more and better results with fewer resources. The primary objective of the Dronfruit project was to create a deep learning-based automated image processing system for identifying, counting, and estimating the size of citrus fruits on individual trees. Twenty trees from a commercial citrus plantation were tracked throughout the course of three yearly campaigns using pictures taken by UAV. Fruit sizes were measured during the hand harvest of these plants. When the estimated and actual yields per tree were compared, the approximation error was found to be SE = 4.53%, and the standard deviation was found to be SD = 0.97 kg. Comparisons were made between the actual total yield, the anticipated total yield, and the total yield calculated by a skilled technician. Whereas the model’s mistakes were SE = 7.22% and SD = 4083.58 kg, the technician’s estimating error was SE = 13.74%^50^.

In our study, the average number of fruits of a *C. unshiu* tress was 842 in August 2021. However, considering the current images were collected in May, which is earlier than normal data collection, and the fruit is detected from the top side, the data that detects more fruit would be close to the actual fruit yield. Therefore, the image from 30 m is more appropriate than the image from 110 m. Also, using the histogram equalized image might be more accurate than using data from the normal image. This study tried vegetation indices (VI) to detect fruits. Vis might be more sensitive to finding fruits than using a histogram equalization image. The I_PCA_ showed similar results to histogram equalization methods, but the I_1_ is not fit for detecting citrus fruits. However, it is possible to roughly predict the number of hidden fruits by various correction coefficients. The results of this experiment confirmed that the sensitivity for finding fruit may vary depending on the type of VI, and the accuracy of yield prediction may also vary depending on post-processing.

The algorithms for fruit recognition based on object detection have limitations since they cannot identify unseen fruits hidden by other fruits or vegetation^51^. Citrus are viewed based on their very changing color, texture, and shape. As a result, while the model cannot recognize all fruits, it can detect the majority of visible fruits. According to Gongal et al., the majority of research on fruit detection has attempted to count the number of fruits per tree as a way to estimate yields^52^.

Efficient and precise crop management requires using high-resolution optical imagery to identify, count, and track individual trees in agricultural settings. Monitoring tree growth, fruit production, and pest and disease occurrence is critical for effective crop management, making the automated delineation of individual trees a valuable tool for long-term management. With the increasing use of UAVs for agricultural applications, there is a growing need for standardized workflows that can help field and research managers effectively integrate large volumes of high-resolution imagery into their management operations. More individual cases are necessary to develop these workflows and improve crop management practices^53–55^. Using flying platforms like UAVs provides a step towards the adoption of this sort of model spanning ever greater regions and at a cheap cost with a system that generates modified results in this manner. Moreover, a new dataset with more photos may be created to provide a better result for yield production prediction. UAVs have recently been increasingly employed as cutting-edge remote sensing platforms for environmental applications^56^. In contrast to field-collected data, UAVs can easily fly over the target region to capture images with extremely high spatial (e.g., centimeters) and temporal (e.g., daily observations) resolutions, which significantly lowers labor and time expenses^57^. The ability to employ a sensor that can be customized on UAVs and the adaptability of altering UAV flying height and attitude can provide us with quick access to data with the spatial and spectral resolutions that customers want^58^. The image is provided with resolutions that are adequately chosen for in-depth observations of crop growth in the field, which is especially advantageous for precision agriculture.

This study developed an approach for computing citrus yield using UAV images. The approach is simple but strongly indicates that spectral mixture analysis should be considered when estimating yield, especially for images that clearly demonstrate the yield of unique spectral components. In order to investigate how resilient our methodology is to variations in climatic factors like temperature, humidity, precipitation, and wind speed, in our future study, we would like to test this method on crops planted in different places under varying weather circumstances.

## Conclusions

The optimal flight altitude depends on the kind of sensor, sensor sensitivity, and the goal of the image used. For instance, a flight altitude of 70 m can enable the optical sensor to achieve a 3D resolution of centimeter-level^5^. However, in this case, 70 m of flight altitude cannot detect fruit accurately. Also, in the other study, the image’s goal was to identify citrus trees; 104 m of the flight altitude was enough to identify citrus trees. However, it is not the appropriate flight height for estimating fruit yield. Considering the UAV image’s goal and the image sensor’s spec in the current study, the 30 m altitude is appropriate, and it might be lower flight height would be better. Using histogram equalization methods increases sensitivity to detect fruit in the images. Besides, it is possible that applying appropriate VIs to detect citrus fruit would be efficient. In this study, Ipca showed good estimation. However, the I_1_ represents inaccurate estimation results. Also, these estimations would increase accuracy with the correlation coefficient. We can easily get data with the spatial and spectral resolutions required by consumers if we can utilize a sensor that can be customized on a UAV and alter the UAV’s flight height and attitude. Precision agriculture benefits significantly from the image’s appropriately selected resolutions for in-depth monitoring of crop growth in the field. In future research, we plan to build an accurate citrus yield prediction model using more UAV sample images, and these technologies and accumulated knowledge can be used not only for yield prediction but also for ground navigation using UAVs.

## Data Availability

The datasets used and analysed during the current study can be available from the corresponding author on reasonable request.

## References

[1] Goldenberg L, Yaniv Y, Porat R, Carmi N (2018). Mandarin fruit quality: A review. J. Sci. Food Agric..

[2] Miles EA, Calder PC (2021). Effects of citrus fruit juices and their bioactive components on inflammation and immunity: A narrative review. Front. Immunol..

[3] Eom HJ, Lee D, Lee S, Noh HJ, Hyun JW, Yi PH, Kang KS, Kim KH (2016). Flavonoids and a limonoid from the fruits of *Citrus unshiu* and their biological activity. J. Agric. Food Chem..

[4] Rafiq S, Kaul R, Sofi SS, Bashir N, Nazir F, Nayik GA (2018). Citrus peel as a source of functional ingredient: A review. J. Saudi Soc. Agric. Sci..

[5] Kimura Y, Naeshiro M, Tominaga Y, Anai T, Komai F (2017). Metabolite composition of grapefruit (*Citrus paradisi*) grown in Japan depends on the growing environment and harvest period. Hortic. J..

[6] Sadka A, Shlizerman L, Kamara I, Blumwald E (2019). Primary metabolism in citrus fruit as affected by its unique structure. Front. Plant Sci..

[7] Zhao XJ, Xing TT, Li YF, Jiao BN (2019). Analysis of phytochemical contributors to antioxidant capacity of the peel of Chinese mandarin and orange varieties. Int. J. Food Sci. Nutr..

[8] Butu M, Rodino S (2019). Fruit and Vegetable-Based Beverages—Nutritional Properties and Health Benefits.

[9] Karunathilake EMBM, Le AT, Heo S, Chung YS, Mansoor S (2023). The path to smart farming: Innovations and opportunities in precision agriculture. Agriculture.

[10] Ku K-B, Mansoor S, Han GD, Chung YS, Tuan TT (2023). Identification of new cold tolerant Zoysia grass species using high-resolution RGB and multi-spectral imaging. Sci. Rep..

[11] Hunt ER, Daughtry CST (2018). What good are unmanned aircraft systems for agricultural remote sensing and precision agriculture?. Int. J. Remote Sens..

[12] Mulla DJ (2013). Twenty five years of remote sensing in precision agriculture: Key advances and remaining knowledge gaps. Biosyst. Eng..

[13] Ali I, Greifeneder F, Stamenkovic J, Neumann M, Notarnicola C (2015). Review of machine learning approaches for biomass and soil moisture retrievals from remote sensing data. Remote Sens..

[14] Carrão H, Russo S, Sepulcre-Canto G, Barbosa P (2016). An empirical standardized soil moisture index for agricultural drought assessment from remotely sensed data. Int. J. Appl. Earth Obs. Geoinf..

[15] Kelly M, Guo Q, Ciancio A, Mukerji KG (2007). Integrated agricultural pest management through remote sensing and spatial analyses. General Concepts in Integrated Pest and Disease.

[16] Peña JM, Torres-Sánchez J, de Castro AI, Kelly M, López-Granados F (2013). Weed mapping in early-season maize fields using object-based analysis of unmanned aerial vehicle (UAV) images. PLoS ONE.

[17] Thorp KR, Tian LF (2004). A review on remote sensing of weeds in agriculture. Precis. Agric..

[18] Lamb DW, Brown RB (2001). PA—Precision agriculture: Remote-sensing and mapping of weeds in crops. J. Agric. Eng. Res..

[19] López-Granados F, Jurado-Expósito M (2006). Using remote sensing for identification of late-season grass weed patches in wheat. Weed Sci..

[20] Berni JAJ, Zarco-Tejada PJ, Suarez L, Fereres E (2009). Thermal and narrowband multispectral remote sensing for vegetation monitoring from an unmanned aerial vehicle. IEEE Trans. Geosci. Remote Sens..

[21] Costes E, Lauri PE, Regnard JL (2006). Analyzing fruit tree architecture: Implications for tree management and fruit production. Hortic. Rev..

[22] Torres-Sánchez J, López-Granados F, Serrano N, Arquero O, Peña JM (2015). High-throughput 3-D monitoring of agricultural-tree plantations with unmanned aerial vehicle (UAV) technology. PLoS ONE.

[23] Lado J, Cuellar F, Rodrigo MJ, Zacarías L (2016). Nutritional Composition of Mandarins.

[24] Fu H, Wang C, Cui G, She W, Zhao L (2021). Ramie yield estimation based on UAV RGB images. Sensors.

[25] Abdulridha J, Batuman O, Ampatzidis Y (2019). UAV-based remote sensing technique to detect citrus canker disease utilizing hyperspectral imaging and machine learning. Remote Sens..

[26] Sheikh M, Farooq IQRA, Ambreen H, Pravin KA, Manzoor IKRA, Chung YS (2023). Integrating artificial intelligence and high-throughput phenotyping for crop improvement. J. Integr. Agric..

[27] López-Granados F, Torres-Sánchez J, Jiménez-Brenes FM, Arquero O, Lovera M, de Castro AI (2019). An efficient RGB-UAV-based platform for field almond tree phenotyping: 3-D architecture and flowering traits. Plant Methods.

[28] Alquezar B, Rodrigo MJ, Zacarías L (2008). Regulation of carotenoid biosynthesis during fruit maturation in the red-fleshed orange mutant Cara Cara. Phytochemistry.

[29] Kato M, Ikoma Y, Matsumoto H, Sugiura M, Hyodo H, Yano M (2004). Accumulation of carotenoids and expression of carotenoid biosynthetic genes during maturation in citrus fruit. Plant Physiol..

[30] Rodrigo M-J, Marcos JF, Zacarías L (2004). Biochemical and molecular analysis of carotenoid biosynthesis in flavedo of orange (*Citrus sinensis* L.) during fruit development and maturation. J. Agric. Food Chem..

[31] Dorj U-O, Lee M, Yun S-S (2017). An yield estimation in citrus orchards via fruit detection and counting using image processing. Comput. Electron. Agric..

[32] Ali A, Imran M (2021). Remotely sensed real-time quantification of biophysical and biochemical traits of Citrus (*Citrus sinensis* L.) fruit orchards—A review. Sci. Hortic..

[33] Park S-H, Kim M-J, Kwon SH, Kim JH, Kang S-B, Park Y-S, Mo C (2022). A study on spectral characteristics of citrus trees in the field using hyperspectral imaging-based drone. Korean Soc. Agric. Mach..

[34] Hsu W-Y, Chou C-Y (2015). Medical image enhancement using modified color histogram equalization. J. Med. Biol. Eng..

[35] Maldonado W, Barbosa JC (2016). Automatic green fruit counting in orange trees using digital images. Comput. Electron. Agric..

[36] Kim, C. S. H. & Min, J. *Investigation on the Fruiting of Citrus Fruits*. https://www.farmnmarket.com/news/article.html?no=18024 (2022).

[37] Sánchez-Sastre LF (2020). Assessment of RGB vegetation indices to estimate chlorophyll content in sugar beet leaves in the final cultivation stage. AgriEngineering.

[38] Acharya MC, Thapa RB (2015). Remote sensing and its application in agricultural pest management. J. Agric. Environ..

[39] Csillik O, Cherbini J, Johnson R, Lyons A, Kelly M (2018). Identification of citrus trees from unmanned aerial vehicle imagery using convolutional neural networks. Drones.

[40] Surový P, Almeida Ribeiro N, Panagiotidis D (2018). Estimation of positions and heights from UAV-sensed imagery in tree plantations in agrosilvopastoral systems. Int. J. Remote Sens..

[41] Anderson K (2013). Gaston Lightweight unmanned aerial vehicles will revolutionize spatial ecology. Front. Ecol. Environ..

[42] Kislik C, Dronova I, Kelly M (2018). UAVs in support of algal bloom research: A review of current applications and future opportunities. Drones.

[43] Zhang C, Kovacs JM (2012). The application of small unmanned aerial systems for precision agriculture: A review. Precis. Agric..

[44] Honkavaara E, Saari H, Kaivosoja J, Pölönen I, Hakala T, Litkey P, Mäkynen J, Pesonen L (2013). Processing and assessment of spectrometric, stereoscopic imagery collected using a lightweight UAV spectral camera for precision agriculture. Remote Sens..

[45] Gonzalez-Dugo V, Zarco-Tejada P, Nicolás E, Nortes PA, Alarcón JJ, Intrigliolo DS, Fereres E (2013). Using high resolution UAV thermal imagery to assess the variability in the water status of five fruit tree species within a commercial orchard. Precis. Agric..

[46] Sankaran S, Khot LR, Espinoza CZ, Jarolmasjed S, Sathuvalli VR, Vandemark GJ, Miklas PN, Carter AH, Pumphrey MO, Knowles NR (2015). Low-altitude, high-resolution aerial imaging systems for row and field crop phenotyping: A review. Eur. J. Agron..

[47] Wahab I, Hall O, Jirström M (2018). Remote sensing of yields: Application of UAV imagery-derived NDVI for estimating maize vigor and yields in complex farming systems in Sub-Saharan Africa. Drones.

[48] Maresma Á, Ariza M, Martínez E, Lloveras J, Martínez-Casasnovas J (2016). Analysis of vegetation indices to determine nitrogen application and yield prediction in maize (*Zea mays* L.) from a standard UAV service. Remote Sens..

[49] Singh AP (2022). A bibliometric review of the use of unmanned aerial vehicles in precision agriculture and precision viticulture for sensing applications. Remote Sens..

[50] http://sparkle-project.eu/dronfruit-project-yield-estimations-in-citrus-orchards-via-drones-and-computer-vision/.

[51] Kamilaris A, Prenafeta-Boldú FX (2018). Deep learning in agriculture: A survey. Comput. Electron. Agric..

[52] Gongal A (2015). Sensors and systems for fruit detection and localization: A review. Comput. Electron. Agric..

[53] Tatsumi K, Igarashi N, Mengxue X (2021). Prediction of plant-level tomato biomass and yield using machine learning with unmanned aerial vehicle imagery. Plant Methods.

[54] Liakos K, Busato P, Moshou D, Pearson S, Bochtis D (2018). Machine learning in agriculture: A review. Sensors.

[55] Elarab M, Ticlavilca AM, Torres-Rua AF (2015). Estimating chlorophyll with thermal and broadband multispectral high resolution imagery from an unmanned aerial system using relevance vector machines for precision agriculture. Int. J. Appl. Earth Obs. Geoinf..

[56] Berni JAJ, Zarco-Tejada PJ, Suárez L (2009). Thermal and narrowband multispectral remote sensing for vegetation monitoring from an unmanned aerial vehicle. IEEE Trans. Geosci. Remote Sens..

[57] Du M, Noguchi N (2017). Monitoring of wheat growth status and mapping of wheat yield’s within-field spatial variations using color images acquired from UAV-camera system. Remote Sens..

[58] Holman F, Riche A, Michalski A (2016). High throughput field phenotyping of wheat plant height and growth rate in field plot trials using UAV based remote sensing. Remote Sens..

