# Author Correction: Enhancing citrus fruit yield investigations through flight height optimization with UAV imaging

**DOI:** 10.1038/s41598-024-57573-2

**Published:** 2024-03-26

**Authors:** Soon-Hwa Kwon, Ki Bon Ku, Anh Tuan Le, Gyung Deok Han, Yosup Park, Jaehong Kim, Thai Thanh Tuan, Yong Suk Chung, Sheikh Mansoor

**Affiliations:** 1 Citrus Research Institute, National Institute of Horticultural and Herbal Science, Rural Development Administration, Jeju, 63607 Republic of Korea; 2 Department of Plant Resources and Environment, Jeju National University, Jeju, 63243 Republic of Korea; 3 Department of Practical Arts Education, Cheongju National University of Education, Cheongju, 28690 Republic of Korea

Correction to: *Scientific Reports* 10.1038/s41598-023-50921-8, published online 03 January 2024

In the original version of this Article, Yong Suk Chung was omitted as a corresponding author. Correspondence and requests for materials should also be addressed to yschung@jejunu.ac.kr.

The original Article has been corrected.

